# Development of a Radial Pulse Tonometric (RPT) Sensor with a Temperature Compensation Mechanism

**DOI:** 10.3390/s130100611

**Published:** 2013-01-04

**Authors:** Seong-Ki Yoo, Ki-Young Shin, Tae-Bum Lee, Seung-Oh Jin, Jaeuk U. Kim

**Affiliations:** 1 Advanced Medical Device Center, Korea Electrotechnology Research Institute, 111, Hanggaul-ro, Sangnok-gu, Ansan, Gyeonggi-do, 426-910, Korea; E-Mails: sky10@keri.re.kr (S.-K.Y.); metode27@keri.re.kr (T.-B.L.); sojin@keri.re.kr (S.-O.J.); 2 Korea Institute of Oriental Medicine, 486, Expo-ro, Yuseong-gu, Daejeon, 305-811, Korea; E-Mail: jaeukkim@kiom.re.kr

**Keywords:** pulse diagnosis, FEM, pressure sensor, thermistor, temperature compensation

## Abstract

Several RPT sensors have been developed to acquire objective and quantitative pulse waves. These sensors offer improved performance with respect to pressure calibration, size and sensor deployment, but not temperature. Since most pressure sensors are sensitive to temperature, various temperature compensation techniques have been developed, but these techniques are largely inapplicable to RPT sensors due to the size restrictions of the sensor, and incompatibility between the compensation techniques and the RPT sensor. Consequently, in this paper a new RPT sensor comprising six piezoresistive pressure sensors and one thermistor has been developed through finite element analysis and then a suitable temperature compensation technique has been proposed. This technique compensates for temperature variations by using the thermistor and simple compensation equations. As verification of the proposed compensation technique, pulse waves of all types were successfully compensated for temperature changes.

## 1. Introduction

Pulse diagnosis is a representative diagnostic method, which, among the four diagnostic methods of observation, listening/smelling, inquiring and palpation described in Traditional Chinese Medicine (TCM) and Korean Medicine (KM), belongs to the palpation class [1]. Pulse diagnosis determines a patient's physical condition, evolution of a disease, causes of a disease, whereabouts of a disease, and a cure for the disease, according to the subjective sense, feeling and experience of the oriental medicine doctor. Thus a pulse diagnosis can be different, according to the conditions of the doctor of oriental medicine or the particular oriental doctor, although pulse diagnosis is traditional and venerable. In order to overcome this problem, objectification of the pulse diagnosis technique is essential [2,3]. The key point for the objectification of pulse diagnosis is to acquire quantitative and objective pulse waves from the radial artery. To achieve this, several types of Radial Pulse Tonometric (RPT) sensors comprising pressure sensors [4–6] and pulse diagnosis instruments [7,8] have been studied and developed. In general, a pressure sensor is adopted to acquire pulse waves [9–11]. The representative types of pressure sensor are the strain gauge, semiconductor piezoresistive pressure sensor, polyvinylidene fluoride (PVDF) piezoelectric pressure sensor, *etc.* [12–14].

In the development of a RPT sensor, the following features should be considered: size, sensor deployment and temperature. The size of the RPT sensor should be appropriate to measure pulses from the radial artery [4,5], because the normal width of this blood vessel is only 2.5 mm [15,16]. If the size of the RPT sensor is too large, it is obstructed from accurately measuring the pulse waves by both the radius and the tendons on the wrist. Conversely, if the sensor is too small, it is unable to satisfactorily cover the radial artery. Pressure sensors have been deployed in a 3 × 3 multi array [6] and linear array [4,5]. The 3 × 3 multi array improves the durability of the sensor, and minimizes crosstalk between pressure sensors, but it is unable to measure pulse waves properly, unless the center of the RPT sensor is located at the point where pulsation is the greatest. The linear array, on the other hand, can easily measure pulse waves. This is because the linear sensor array is placed vertically on the radial artery to measure the pulse waves and there is no need to place a RPT sensor at an exact position. It only needs to be approximately located between the radius and the tendon. Thus, it is more stable for measuring pulse waves. These RPT sensors have been considered and improved with respect to size and sensor deployment, but not temperature.

Most pressure sensors are affected by temperature. For this reason, various temperature compensation techniques have been developed and employed to improve the measurement accuracy of pressure sensors [17–22]. These temperature compensation methods can be divided into two categories: hardware-based [17–19], and those that use a combination of hardware and software [20–22].

In the hardware method, a compensation circuit is generally used for temperature compensation. For example, a Proportional To Absolute Temperature (PTAT) circuit is employed to compensate for temperature variations by taking advantage of the positive linear behavior of PTAT [17]. Furthermore, the double-bridge compensation technique and temperature half-bridge compensation technique are also employed for temperature compensation. [19]. These hardware methods are not complicated, so they can be easy and fast to implement. Moreover, these methods can be applicable over a wide temperature range. Despite these advantages, however, these various hardware-based compensation techniques have problems, such as the size restrictions of the RPT sensor, and incompatibility in applying them to the RPT sensor. If a compensation circuit is added to a RPT sensor, the size of the bigger RPT sensor will now influence measurement of the radial artery pulses, due to obstruction from both the radius and tendon on the wrist. This implies that the measurement accuracy of the RPT sensor is degraded by the size of RPT sensor. Moreover pressure sensors in the fabricated RPT sensors are bonded on a Printed Circuit Board (PCB) substrate by the die-bonding method, thus it is difficult to connect additional components with the active arms of a Wheatstone bridge in series or parallel. That is, temperature compensation techniques that require connection of additional components with a Wheatstone bridge are inapplicable to a RPT sensor.

A single example of the combination of hardware and software methods is an online temperature compensation scheme using an Artificial Neural Network (ANN) [20]. This method corrects temperature sensitivity by using an on-board memory. The use of an ANN for compensation of the temperature errors of a signal conditioning circuit is a well-known technique. Another example uses a MAX1452, and a curve fitting algorithm based on a cubic B-spline, which are employed in the hardware and in the software design, respectively [21]. These methods are stable, and greatly improve the efficiency and accuracy of the silicon piezoresistive pressure sensor, thanks to the harmony between hardware and software. However, an online temperature compensation scheme using ANN costs a considerable amount of money to the build the necessary hardware for parallel processing. Moreover, it requires lengthy training times for operation. The curve fitting algorithm based on a cubic B-spline requires complex computations for a large set of control points, so it takes a comparatively long time to implement a compensation technique. Due to these drawbacks, these methods are also not applicable to a RPT sensor.

The purpose of this paper was to develop a RPT sensor by considering not only the size, and sensor deployment, but also temperature variation. Nowadays, the Finite Element Method (FEM) is a common tool for modeling of the structure of a sensor. Therefore, the new RPT sensor presented in this paper was fabricated based on FEM simulations: a polydimethylsiloxane (PDMS) thickness-dependent simulation, and a temperature distribution simulation. The fabricated sensor module comprises pressure sensors and a thermistor. The temperature of a RPT sensor becomes stable at a specific temperature which is the ambient temperature, but when a RPT sensor is placed on the wrist for measurement of pulse waves, however, the temperature of the sensor is changed, and it may take several seconds or minutes for the temperature of a RPT sensor to become stable again. For this reason, temperature compensation of a RPT sensor is important to acquire quantitative and objective pulse waves. Therefore the thermistor monitors and measures the temperature of a RPT sensor in real time, while measuring the pulse waves of the radial artery. Then the measured pulse waves are compensated for temperature variations based on the temperature measured by the thermistor.

## 2. Materials and Methods

### 2.1. FEM Modeling: Pressure Distribution According to PDMS Thickness

Silicon is usually employed for sensor packaging, due to its outstanding mechanical properties. Among the various types of silicon, PDMS is transparent, elastic and cheap. In addition, PDMS has the properties of being easy to cast and mold. For this reason, PDMS is employed in various industrial fields, including sensors [23,24]. According to the properties and thickness of the material used in sensor packaging, the pressure transferred to the RPT sensor and the degree of influence on the surrounding area can be changed. These changes can cause errors in measuring and restoring bio-signals. In order to analyze the influence of sensor packaging on sensor operation, therefore, many studies using FEM have been conducted [25].

**Figure 1.**
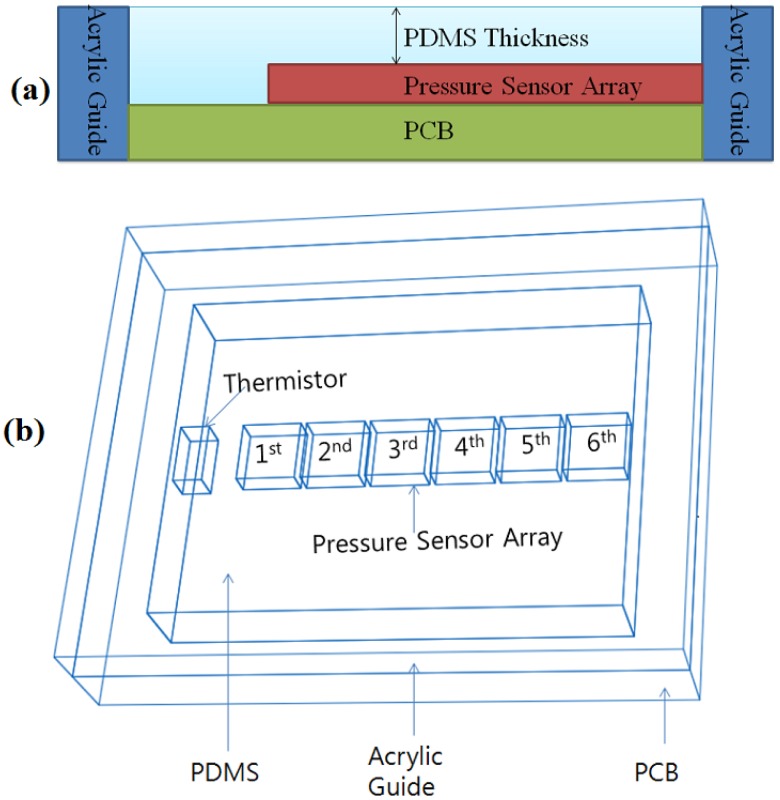
Scenes of pressure drop and influence on surrounding area simulation: (**a**) definition of PDMS thickness, (**b**) shape information and composition of a RPT sensor.

In this study, PDMS (Sylgard 184, Dow Corning) was employed as the material of a RPT sensor packaging. The use of PDMS inevitably influences the enclosed sensors. Thus, Abaqus 6.71 was used to analyze the pressure distribution and influence on the surrounding area, according to PDMS thickness. Three thicknesses of PDMS are used for simulation: 0.8, 1.0 and 1.2 mm. Please note that the PDMS thickness refers to the height from the pressure sensor to the upper side of the PDMS, as shown in Figure 1(a), not from the base side to the upper side of the PDMS. Figure 1(b) shows the shape information and composition of a RPT sensor. For this simulation, a pressure of 100 Pa is loaded onto the 3rd pressure sensor.

### 2.2. FEM Modeling: Temperature Distribution of the RPT Sensor under Different Temperatures

When the pulse waves of patients are measured by a RPT sensor, heat conduction occurs due to the temperature difference between the skin temperature and the surface temperature of the RPT sensor. For example, the surface temperature of a RPT sensor is increased when the surface temperature of a RPT sensor is lower than the skin temperature. This temperature change interferes with the acquisition of an objective pulse wave. This interference causes a distortion of the pulse wave. In this simulation, therefore, the temperature distribution when a certain heat was applied on the upper side of the RPT sensor was studied. For the simulation, the upper side of the RPT sensor is set at 36.5 °C. In addition, both the side face and the base side of the RPT sensor are set at the surface temperature of the RPT sensor, which is the same as room temperature. This is because the temperature of the RPT sensor is stable at ambient temperature. In order to find the temperature distribution according to different room temperatures, three different room temperatures are used: 20, 30, and 40 °C.

### 2.3. Design of the RPT sensor

Our proposed RPT sensor consists of six piezoresistive pressure sensors (C33 series, EPCOS, Munich, Germany), and one Negative Temperature Coefficient (NTC) thermistor (NCP15XH103DRA, Murata, Tokyo, Japan), to measure pulse waves and the surface temperature of the RPT sensor, respectively. The piezoresistive pressure sensor is configured in a Wheatstone bridge, with four active arms. The size of the pressure sensor is 1.00 mm × 1.00 mm, and mV outputs are generated, according to the applied pressure. A NTC thermistor of size 1.00 × 0.50 mm has 10 kΩ resistance at 25 °C. When the temperature increases, the value of the resistance decreases. These pressure sensors and the NTC thermistor are electrically connected with the PCB by wire-bonding, and Surface Mounting Technology (SMT), respectively. A PDMS coating is formed on the elements of a RPT sensor to protect the fragile pressure sensors, thermistor and wires from the direct pressure applied to the sensors. Moreover, an acrylic guide is bonded on the PCB to maintain a constant height of the PDMS coating, and to prevent damage to the edges of the RPT sensor. The size of the designed RPT sensor is 10 × 8 × 2 mm.

### 2.4. Calibration of the RPT Sensor and Temperature Compensation

**Figure 2.**
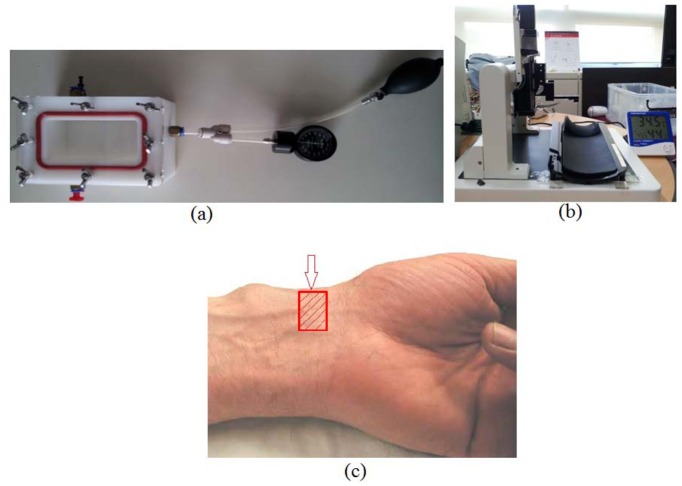
Equipment: (**a**) Pressure chamber with an air-pressure pump and a pressure gauge, (**b**) Test jig for verification of the temperature compensation technique, (**c**) A RPT sensor is placed on the radial artery vertically.

In order to establish our temperature compensation technique, the output characteristics of a RPT sensor at different pressures and temperatures need to be evaluated. For this reason, a pressure chamber, as shown in Figure 2(a) with the size of 165 mm × 115 mm × 95 mm, and a steady temperature and humidity room equipped with a thermohygrostat (ZEPHYRUS, Shinsung Engineering, Seoul, Korea) were used to maintain constant pressure and temperature, respectively. This pressure chamber is connected with an air-pressure pump and a pressure gauge, to apply and measure the pressure in the chamber. A RPT sensor is put into the pressure chamber, which is used to apply pressure to the RPT sensor. Then, the RPT sensor outputs are measured at eight different pressure steps, from 0 mmHg to 210 mmHg, with intervals of 30 mmHg, at 20, 30 and 40 °C. Moreover, a Data Acquisition (DAQ) board (NI USB-6210) was employed to acquire the outputs of the RPT sensor. The RPT sensor output, including the pressure sensor and thermistor outputs, are measured for 5 seconds, at a sample rate of 400 Hz. Note that pressure is applied by the air-pump when the temperature in the pressure chamber is the same as the temperature of the steady temperature and humidity room. In order to check the temperature in the pressure chamber, the output of the thermistor in the RPT sensor is used. However, the thermistor output is a voltage, not temperature, therefore, the output voltage of the thermistor needs to be converted into temperature units. For this, the output of the thermistor was measured from 20 °C to 40 °C, at 5 °C intervals, to determine the output characteristics of the thermistor according to temperature changes. From these measurements, a linear equation can be obtained and the temperature inside the pressure chamber can be determined by using this linear equation. In addition, a digital thermometer was used to check that the room temperature was the same as the desired temperature as controlled by the thermohygrostat.

To verify the proposed compensation equation, pulse waves of six subjects were collected at 20 °C, 30 °C and 40 °C. This experiment was conducted in a steady temperature and humidity room equipped with a thermohygrostat so as to maintain the temperature constant. All six subjects were male and healthy. The average age, height and weight of the six subjects were 34.3 ± 4.0 years old, 175.7 ± 4.3 cm, and 72.7 ± 11.4 kg, respectively. The outputs of the six pressure sensors and the thermistor in the RPT sensor are measured at a sampling rate of 100 Hz for 2 minutes by the DAQ board (NI USB-6210). In order to study the temperature changes at 20 °C, 30 °C and 40 °C, the stable skin temperature is subtracted from the initial skin temperature. In addition, the compensated pulse wave was subtracted from the uncompensated pulse wave to study the pressure difference between them. In this verification experiment, two RPT sensors (sensors A and B) are used to discuss the correlation of two RPT sensors. RPT sensor B was used after the pulse wave of one subject was measured by RPT sensor A. Then another subject followed the same procedure. Please note that each subject waited for 5 min in the steady temperature and humidity room until his skin temperature became stable. For the same reason, the RPT sensor A should not be used again for 5 min after being used. The test jig shown in Figure 2(b) was employed to measure pulse waves. This test jig was equipped with a RPT sensor module and a stepping motor. The RPT sensor is moved up and down by a stepping motor. The RPT sensor stops moving, and pulse waves are saved automatically when the detected pressure on the RPT sensor is over the threshold (e.g., 80 mmHg). This automatic system is controlled by a Labview program. Note that the RPT sensor moves and measures pulse waves when the temperature detected by the thermistor, a digital thermometer, and the desired temperature of the thermohygrostat are all the same.

## 3. Results

### 3.1. Computer Simulation: Load Pressure Distribution According to PDMS Thickness

**Figure 3.**
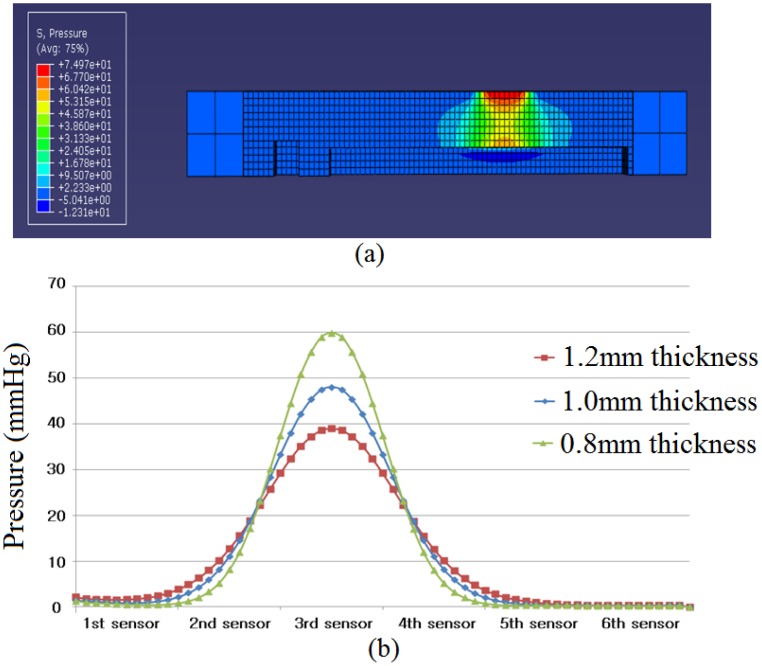
Results of computer simulation: (**a**) load pressure distribution in cross-section view of RPT sensor, (**b**) pressure of the center of pressure sensors according to PDMS thickness.

Figure 3(a) shows the pressure distribution when a pressure of 100 Pa was applied to the 3rd pressure sensor. It is easy to see that the applied pressure decreased through the PDMS. In this simulation the pressure of 100 Pa was only applied to the 3rd pressure sensor, but it was distributed to the surrounding pressure sensors. For an accurate analysis, the distributed pressures on the center points of the pressure sensors were recorded and plotted as shown in Figure 3(b). The maximum pressure was recorded at the 3rd pressure sensor.

In order to study the influence on the surrounding sensors, Table 1 was generated. As the PDMS thickness is increased, pressure on the 3rd sensor is decreased, while pressure on the 2nd sensor is increased. Moreover the influence ratio of the 2nd sensor and the 3rd sensor was minimum when the PDMS thickness was 0.8 mm. From this fact, it can be found that the degree of influence on the surrounding sensor is increased when the PDMS thickness is increased. Then, it can be concluded that among the three PDMS thicknesses tested, 0.8 mm is the most suitable, because 0.8 mm of PDMS thickness has the least degree of influence on the surrounding sensors.

### 3.2. Computer Simulation: Temperature Distribution on the Pressure Sensors and Thermistor

**Figure 4.**
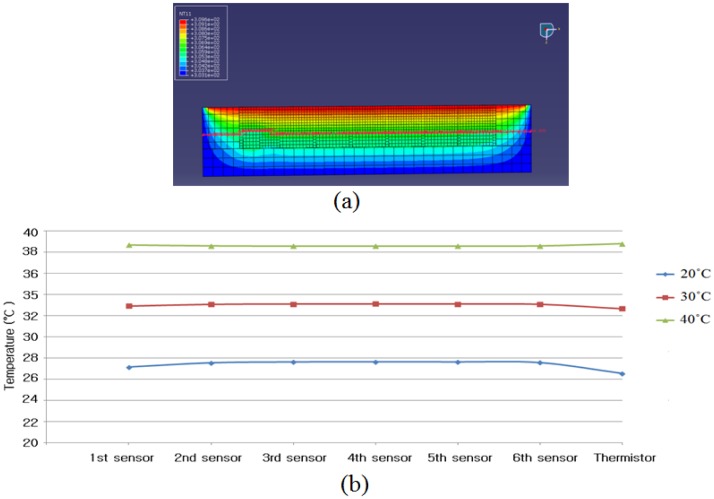
Results of temperature distribution simulation: (**a**) temperature distribution representing cross-section of the RPT sensor, (**b**) temperature on center of each pressure sensor at 20 °C, 30 °C, 40 °C.

Figure 4(a) shows the temperature distribution of the RPT sensor, when the surface temperature of the RPT sensor and skin temperature are 30 °C and 36.5 °C, respectively. The temperature decreases as the distance from the surface of the RPT sensor increases. The temperature of the center point of each pressure sensor and thermistor are recorded. These can be plotted as shown in Figure 4(b). The temperatures detected on the pressure sensors and thermistor are about 27.4 °C, 33 °C, and 38.6 °C, at room temperature of 20 °C, 30 °C, and 40 °C, respectively. It is obvious that the detected temperature on the pressure sensor is different from the room temperature, and the differences between the room temperature and detected temperature are 7.3 °C, 3.0 °C and 1.5 °C at 20 °C, 30 °C and 40 °C, respectively. The biggest difference appears at 20 °C, while the smallest difference appears at 40 °C. It can be induced that there is less temperature difference when the room temperature is closer to the skin temperature.

### 3.3. Fabrication of the RPT sensor

**Figure 5.**
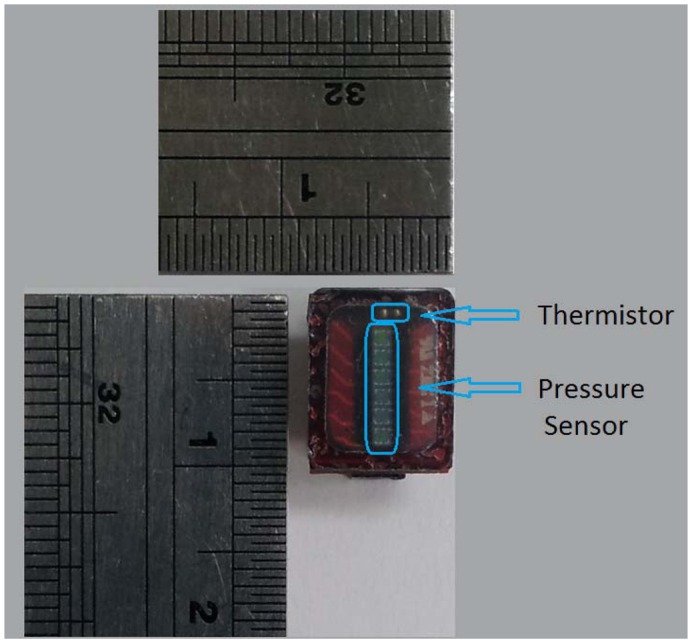
Fabricated RPT sensor.

Figure 5 shows the as fabricated RPT sensor. According to our FEM simulation, the appropriate thickness of PDMS was decided to minimize the effect of PDMS coating in acquiring a pulse wave, and to prevent damage on the edges of the RPT sensor. Moreover, a thermistor was employed to monitor and measure the surface temperature of the RPT sensor for temperature compensation. The actual size of the fabricated RPT sensor is 10 × 8 × 2 mm, which is the same as the designed one.

### 3.4. Establishment and Verification of the Temperature Compensation Technique

**Figure 6.**
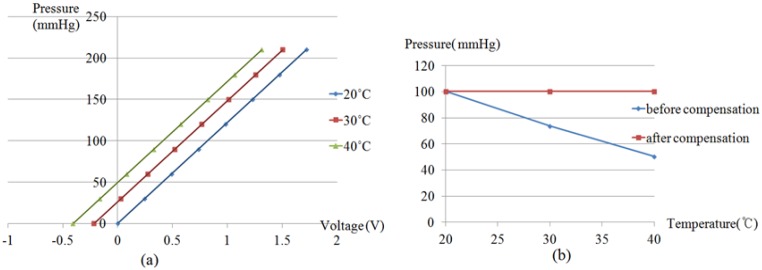
Temperature compensation: (**a**) Voltage-pressure relational equation at 20 °C, 30 °C, 40 °C after offset, (**b**) Comparison simulation of before and after temperature compensation.

The outputs of the RPT sensor at 20 °C, 30 °C and 40 °C were measured, applying pressure from 0 mmHg to 210 mmHg, at 30 mmHg intervals. From these measurements, the relationship among output voltage of the pressure sensors and the applied pressure and temperature can be discovered. The output voltage of a pressure sensor increases when the temperature increases. This implies that the output of a RPT sensor varies according to the temperature. Therefore, temperature compensation is required to provide reliable and accurate pulse waves, regardless of the temperature change. It is easy to see that the output voltage of pressure sensors is increased regularly by a specific voltage. Moreover the rates of increase, according to the applied pressure at 20 °C, 30 °C and 40 °C, are almost the same. Thus, the relationship between the output voltage of a pressure sensor and the applied pressure at any temperature can be expressed as a linear equation. This equation has the same slope and different y-intercepts, according to temperature. In other words, temperature variation can be compensated for by adding or subtracting the corresponding y-intercept to the temperature. In order to calculate the slope and y-intercept, the outputs of pressure sensors at 20 °C, 30 °C and 40 °C are subtracted from the outputs of the pressure sensors at 0 mmHg, 20 °C. Then, the pressure-voltage relational equation can be obtained by the mean of the six pressure sensor measurements. By using a pressure-voltage relational equation, the output voltage can be calculated from the applied pressure. However, a device taking the pulse generally provides pressure as the output, not voltage. Therefore, a new equation need to be generated, called the voltage-pressure relational equation, in which the output voltage of the pressure sensor is plotted along the x-axis, and the applied pressure is plotted along the y-axis. In other words, the inverse version of the pressure-voltage relational equation needs to be found. Figure 6(a) shows the corresponding pressure to the output voltage of a pressure sensor with the pressure-voltage relation equation for each temperature. Now, the RPT sensor is able to provide pressure as output. From Figure 6(a) it can be found that the slope of the pressure-voltage relational equation is 122.15, and the y-intercepts are 0, 26.446 and 49.849 for 20 °C, 30 °C and 40 °C, respectively, in the linear equation model.

**Figure 7.**
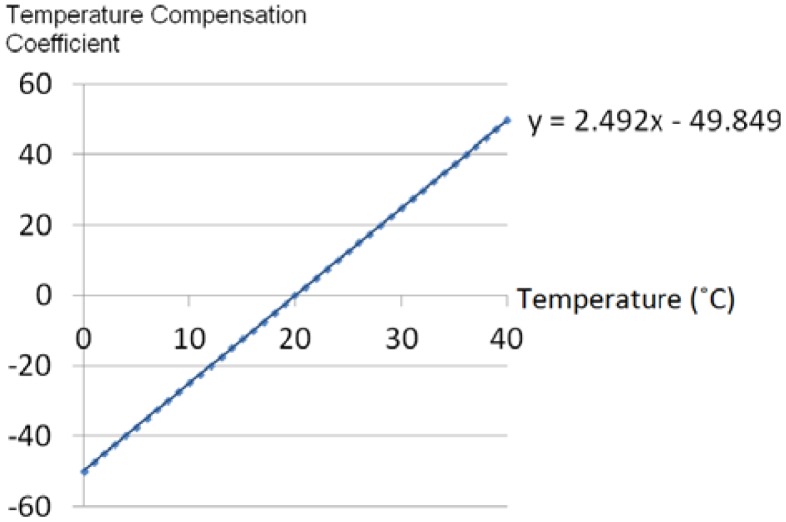
Estimation equation of the temperature compensation coefficient.

Figure 6(b) shows the comparison with and without temperature compensation by computer simulation. It is clear that the proposed compensation method properly compensates for temperature variations. It can be estimated that the y-intercept is increased/decreased by about 2.492 mmHg, every 1 °C increment/decrement. From these factors, the estimation equation for the temperature compensation coefficient can be obtained as shown in Figure 7, and therefore, the temperature compensation coefficient can be estimated by the temperature detected by the RPT sensor.

**Figure 8.**
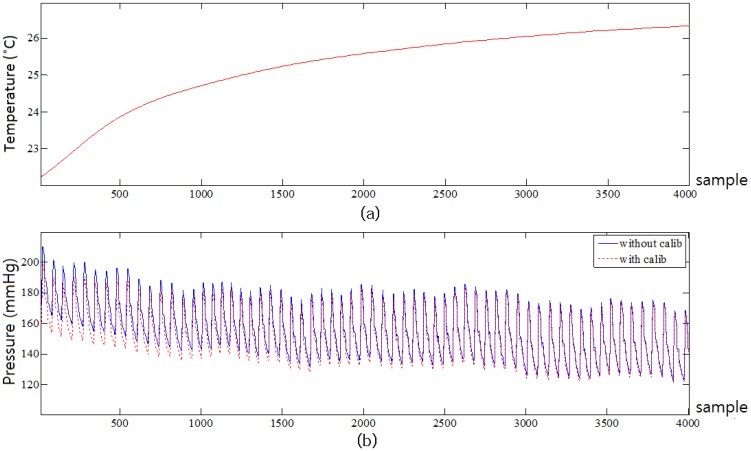
(**a**) Temperature change of the RPT sensor at 20 °C, (**b**) Comparison of compensated and uncompensated pulse wave of one subject at 20 °C.

Figure 8 shows the temperature change of the RPT sensor at 20 °C and the comparison of uncompensated and compensated pulse waves of one subject at 20 °C. It is easy to see that there is rapid temperature change in the beginning of the measurement. In this rapid temperature change period, the difference between uncompensated and compensated pulse wave is larger than in the other periods. On the other hand, the difference between the uncompensated and compensated wave becomes small as the temperature of the RPT sensor becomes stable. The correlation between two RPT sensors was calculated to describe the degree of relationship between two RPT sensors. The correlation coefficients at 20 °C, 30 °C and 40 °C were 0.9999, 0.9999 and 0.9992, respectively. They are all larger than 0.999 at each temperature, which implies that correlation between two RPT sensors is high.

When the RPT sensor is exposed to the skin, the initial skin temperature and stable skin temperature of each subject were different. However the skin temperature changes of each subject were similar as shown in Table 2. Tables 2 and 3 represent the surface temperature changes of the RPT sensor and the pressure difference between compensated and uncompensated pulse waves of all subjects, respectively, measured by two RPT sensors at 20 °C, 30 °C and 40 °C. The average temperature change at 20 °C, 30 °C and 40 °C is about 4.6 °C, 1.1 °C and 1.9 °C, respectively. It is easy to see that the largest temperature change occurs at 20 °C. The mean difference between compensated and uncompensated pulse waves at 30 °C and 40 °C are about 2 mmHg and 4 mmHg, respectively, whereas the mean difference at 20 °C is about 10 mmHg. This implies that the difference between compensated and uncompensated pulse waves is increased, when the temperature change of the RPT sensor is increased. This temperature change is determined by the difference between surface temperature of RPT sensor and skin temperature. If the allowable pressure error is assumed as 5 mmHg, the mean difference at 30 °C and 40 °C can be negligible, whereas the mean difference at 20 °C is too large to be considered negligible. Therefore, temperature compensation is required for accurate pulse waves, when room temperature is close to 20 °C.

## 4. Discussion and Conclusions

In general, a pressure sensor is adopted to acquire pulse waves. It is essential to acquire quantitative and objective pulse waves for the objectification of pulse diagnosis. In practice, however, it is difficult to acquire accurate pulse waves, due to several factors. One of these factors is temperature, as pressure sensors are sensitive to ambient temperature changes. The temperature of a RPT sensor stabilizes at a specific temperature determined by the ambient temperature. When a RPT sensor is placed on the wrist for measurement of pulse waves, however, the temperature of the sensor is changed, and it can take several seconds or minutes for the temperature of a RPT sensor to become stable again. This is not a short time, considering that one of disadvantages of a pulse diagnosis instrument is that it is time-consuming. For this reason, temperature compensation of a RPT sensor is important to acquire quantitative and objective pulse waves. In this paper, therefore, a new RPT sensor has been developed through FEM analysis. We analyzed the pressure distribution and influence of the surrounding sensor according to PDMS thickness. According to the simulation results, pressure signal loss and the degree of influence on neighboring sensors are decreased as PDMS thickness is decreased. In this paper, 1.0 mm of PDMS thickness was determined. The performance of the RPT sensor can be improved if the PDMS thickness is less than 1.0 mm, but there is a limitation in how much the PDMS height can be decreased, due to manufacturing process limitations, and the structural problems of connecting the pressure sensors and PCB with wires. A study to overcome these limitations will be conducted later. Moreover, the temperature distribution of a RPT sensor was analyzed.

The developed RPT sensor consists of six pressure sensors and one thermistor, and the proposed technique compensates for temperature variations by using the thermistor and an equation for estimation of the appropriate temperature compensation coefficient. These temperature compensation coefficient estimation equations were derived through experiments, in which the output of the RPT sensor inside a pressure chamber was measured over eight different pressure steps, from 0 mmHg to 210 mmHg, at 30 mmHg intervals, at 20 °C, 30 °C and 40 °C. To verify the proposed compensation technique, pulse waves of six subjects were measured at 20 °C, 30 °C and 40 °C. The surface temperature changes of the RPT sensor at 20 °C, 30 °C and 40 °C were about 4.6 °C, 1.1 °C and 1.9 °C, respectively. This is because 20 °C represent the biggest difference from the skin temperature. This implies that surface temperature change of a RPT sensor is increased as the difference between the skin temperature and room temperature is increased. However, it was found that the difference between room temperature and detected temperatures on pressure sensors at 20 °C, 30 °C and 40 °C were 7.3 °C, 3.0 °C and 1.5 °C, respectively, in the temperature distribution simulation. Although the values of the differences were somewhat different, it was also concluded that the biggest difference appears at 20 °C. The skin temperature might be affected by room temperature. This means that skin temperature is not exactly 36.5 °C. However, 36.5 °C was used as the skin temperature in our FEM simulation. This error is one reason why the values of temperature difference are different. Another reason is that the skin temperature varies from person to person.

The pressure difference between compensated and uncompensated pulse waves at 30 °C and 40 °C are about 2 mmHg and 4 mmHg, respectively, whereas the difference at 20 °C is about 10 mmHg. This means that the error at 20 °C is relatively larger than the ones at 30 °C and 40 °C. With the assumption that the allowable pressure error is 5 mmHg, the difference between compensated and uncompensated pulse waves could be ignored at 30 °C and 40 °C, whereas the difference between compensated and uncompensated pulse waves at 20 °C was too large to be ignored. The optimal room temperature in summer is between 26 °C and 28 °C, while that in winter it is between 18 °C and 20 °C, so the optimal room temperatures in summer and in winter are close to 30 °C and 20 °C, respectively. Thus, considering the optimal room temperature, temperature compensation might not be required in summer. This depends on the value of the allowable pressure error. In winter, however, that temperature compensation technique is required to acquire accurate pulse waves from patients.

The compensation technique proposed in this paper is a combination of hardware (a thermistor) and software (of temperature compensation coefficient estimation equations). In comparison with other methods, this compensation technique is simple and cheap to implement. In general, an additional component and circuit are required for temperature compensation. However, if the RPT sensor temperature can be measured by means of the sensor itself, without any other components such as a thermistor, a temperature compensation technique by software alone can be proposed.

In order to employ the developed RPT sensor in a practical pulse diagnosis system, reproducibility and repeatability tests for the verification of the reliability of the RPT sensor are required. The pulse diagnosis system is considered reliable if it is reproduces similar results, when used by several operators to evaluate the same group of patients. In practice, however, the human body displays a wide variability depending on the environment. For this reason, it is difficult to objectively evaluate the repeatability of a RPT sensor, based on the measured pulse waves of subjects. Thus, it is appropriate to use a pulse wave simulator for the evaluation of the repeatability of the sensor, which reproduces the constant pulse wave of a human.

## Figures and Tables

**Table 1.** Ratio of pressure on the 2nd sensor and the 3rd sensor according to PDMS thickness.

**Thickness (mm)**	**Pressure on 2nd Sensor (Pa)**	**Pressure on 3rd Sensor (Pa)**	**2nd/3rd Sensor Ratio (%)**
0.8	8.1140	59.8084	13.57
1.0	11.0628	47.9894	23.05
1.2	12.6833	38.9115	32.59

**Table 2.** Temperature change of a RPT sensor while measuring the pulse wave of six subjects at 20 °C, 30 °C and 40 °C (Unit: °C).

	**Sensor A**			**Sensor B**		

	**20°C**	**30°C**	**40°C**	**20°C**	**30°C**	**40°C**

**Subject 1**	4.96	0.64	1.26	5.54	1.93	1.83
**Subject 2**	4.43	0.37	1.54	4.89	1.51	2.35
**Subject 3**	4.5	0.56	1.33	6.33	2.62	2.71
**Subject 4**	4.28	0.42	2.2	5.76	1.62	1.36
**Subject 5**	4.25	1.83	1.63	4.56	0.97	1.43
**Subject 6**	4.16	0.53	1.83	1.25	0.54	2.96

**Mean**	4.43	0.72	1.63	4.72	1.53	2.11

**Table 3.** Pressure Difference between compensated and uncompensated pulse waves of six subjects at 20°C, 30°C and 40°C (Unit: mmHg).

	**Sensor A**			**Sensor B**		

	**20°C**	**30°C**	**40°C**	**20°C**	**30°C**	**40°C**

**Subject 1**	10.2973	1.048	2.599	2.6462	1.2631	5.7397
**Subject 2**	9.9152	0.9612	3.3995	9.7331	1.983	3.0884
**Subject 3**	9.309	0.7058	2.8263	12.6789	3.3241	3.0146
**Subject 4**	9.1432	0.9444	4.9557	13.4911	5.292	5.996
**Subject 5**	9.2245	3.657	3.55	11.1735	3.2767	5.2576
**Subject 6**	9.1317	1.1528	4.0048	11.8659	3.6037	4.2045

**Mean**	9.50348	1.41153	3.55588	10.2647	3.12376	4.55013
